# Characterizing Granuloma Annulare in 73 Pediatric Patients

**DOI:** 10.1155/2023/9267263

**Published:** 2023-12-09

**Authors:** Ania Stolarczyk, Fatima Bawany, Simon Hernandez, Glynis A. Scott, Maria R. Cordisco

**Affiliations:** ^1^Department of Dermatology, University of Rochester Medical Center, Rochester, NY, USA; ^2^Department of Pathology, University of Rochester Medical Center, Rochester, NY, USA

## Abstract

**Background:**

Granuloma annulare (GA) is a common, benign, idiopathic inflammatory dermatosis. Aside from case reports and small studies, there are limited data about the characteristics of GA in children.

**Objective:**

This study aimed to better characterize the epidemiologic and clinical features, triggering factors, disease associations, and outcomes of GA in the pediatric population.

**Methods:**

We conducted a retrospective study of 73 pediatric patients diagnosed with GA at the University of Rochester Medical Center over a 7-year period.

**Results:**

The most common subtype was localized GA (71.2%, *n* = 52), followed by subcutaneous (also known as “deep GA”; 16.4%, *n* = 12) and generalized (12.3%, *n* = 9) subtypes. Over 90% of patients had idiopathic GA, with the remaining patients reporting viral infection or trauma as triggers. Half of the patients studied had comorbid conditions, most frequently atopic dermatitis (17.8%, *n* = 13), obesity (9.59%, *n* = 7), asthma (6.85%, *n* = 5), and allergic rhinitis (6.85%, *n* = 5). The median duration of the disease was 11.00 months (interquartile range (IQR) 15.75 months); generalized GA had the shortest duration (median 10.00 months, IQR 15.50 months), while subcutaneous GA had the longest duration (median 12.00 months and IQR 29.00 months). Although recurrence rates for subcutaneous and generalized GA were high at 45.5% and 33.3%, respectively, most patients achieved clearance or improvement with treatment.

**Conclusion:**

Most cases of GA in our study were idiopathic, with no clear differences between GA subtypes and associated comorbidities. Topical steroids were the most prescribed treatment with mixed efficacy.

## 1. Introduction

Granuloma annulare (GA) is a benign inflammatory dermatosis of unknown etiology. It is common in children and adults, with an estimated incidence of 0.04% in the United States [1]. Several subtypes of GA exist, including the localized, generalized, subcutaneous, perforating, and patch variants, each with distinct clinical features [2]. In the pediatric population, the localized and subcutaneous subtypes are the most common [3].

Several aspects of GA remain poorly understood. Its association with other diseases, for example, remains heavily debated. The association with diabetes mellitus has been particularly controversial, with studies yielding conflicting results [4–6]. Very little data exist regarding associated diseases in pediatric patients or between various GA subtypes. There are also limited data on efficacious treatments for GA. Generalized GA, which has been historically difficult to manage, has had several proposed treatments with mixed results largely based on case reports and small retrospective studies [7].

Given the paucity of studies characterizing GA in the pediatric population, we are presenting this study to review the epidemiologic and clinical features, triggering factors, disease associations, and outcomes of GA in pediatric patients.

## 2. Methods

### 2.1. Study Design

We conducted a retrospective single-center study at the Pediatric Dermatology Clinic in the University of Rochester Medical Center (URMC) Department of Dermatology in Rochester, NY. This study was approved by the Institutional Review Board (IRB) at the University of Rochester Medical Center. Inclusion criteria included age <18 years at the time of GA diagnosis, an ICD-10 diagnosis of “granuloma annulare” (L92.0) in the EPIC electronic medical record, and presentation to URMC between January 1, 2014, and December 20, 2020.

### 2.2. Data Collection and Analysis

Once inclusion criteria were met, a retrospective electronic medical record chart review was conducted to obtain information on patient demographics (gender, ethnicity/race, age of GA onset, and GA evaluation), medical history, family history, known triggers, clinical and histological findings, treatments used, and disease course. All patient data were deidentified and recorded in a secure spreadsheet. Statistical analyses were performed using the GraphPad Prism Software Version 9.3.1. Descriptive statistics were used for demographic variables including sex, age, and race. Quantitative variables were expressed as mean ± standard deviation (SD), whereas qualitative variables were expressed in frequencies. The duration was expressed as median ± interquartile range (IQR).

## 3. Results

Table 1 describes the demographics of our study cohort and clinical features of patients' disease. Our cohort consisted of 73 patients, most of whom were female (63.0%, *n* = 46) and Caucasian (53.4%, *n* = 39). The mean age of GA onset was 6.21 years (standard deviation (SD) 4.34 years).

Approximately 28.8% (*n* = 21) had a biopsy supporting their diagnosis. The most common subtype was localized GA (71.2%, *n* = 52), followed by subcutaneous (16.4%, *n* = 12) and generalized (12.3%, *n* = 9) subtypes. No patients in our cohort were diagnosed with patch or perforating GA subtypes.

**Figure 1:**
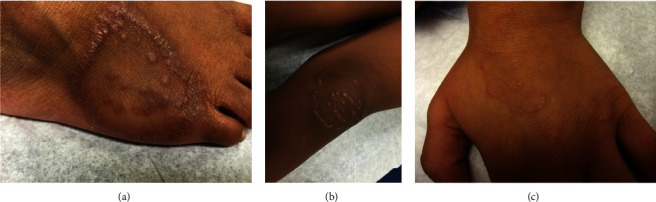
Localized granuloma annulare presenting as erythematous to violaceous annular plaques on the foot (a), knee (b), and dorsal hand (c) on Fitzpatrick skin types IV through V.

**Figure 2:**
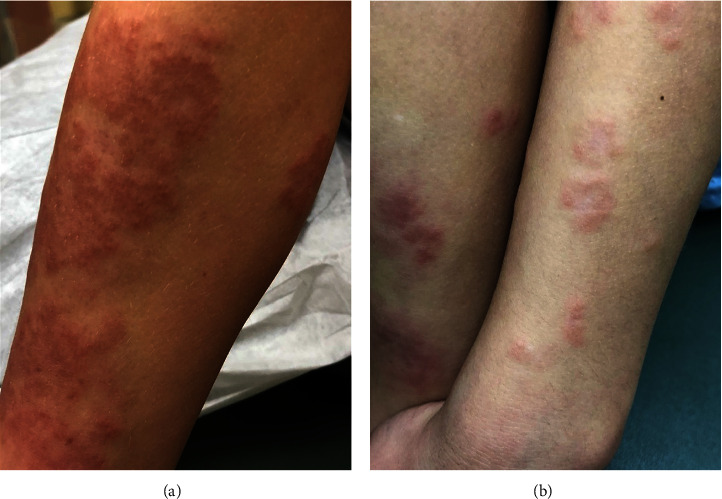
Generalized granuloma annulare presenting as erythematous papules in a circinate pattern on the extensor surfaces of the leg (a) and the arm with truncal involvement (b).

**Figure 3:**
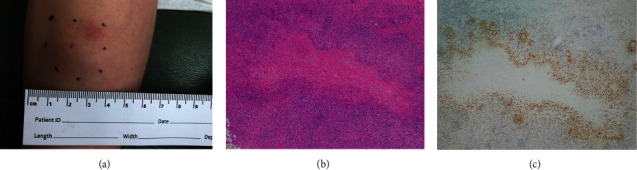
Biopsy site of deep granuloma annulare presenting as a solitary, firm nodule in the pretibial region (a). Histology shows (b) prominent necrobiotic collagen with palisading histiocytes and scattered lymphocytes, characteristic of deep granuloma annulare (H&E, 4X), and (c) immunocytochemical stains against CD68 highlight the histiocytes in the infiltrate (4X).

Localized GA commonly presented on the arms, legs, hands, or feet with multiple asymptomatic papules in an annular or circinate pattern mainly located on the extensor surfaces of the lower and upper extremities, with a minority of patients experiencing symptoms of pruritus (23.1%, *n* = 12) or pain (3.85%, *n* = 2) (Figure 1). Generalized GA had a similar morphology, but typically involved the trunk in addition to the extremities, and had associated pruritus in less than half of cases (44.4%, *n* = 4) (Figure 2). Finally, the subcutaneous subtype was commonly a solitary, firm, nonulcerated, nontender, and sometimes pinkish nodule. The most common location was on the lower extremities, especially the pretibial area, followed by the hands (Figure 3(a)). Buttocks, scalp, and forehead were less commonly affected. The nodules were symptomatic in 41.7% of the cases (*n* = 5), notable for symptoms of pruritus (33.3%, *n* = 4) or pain (8.33%, *n* = 1).

Most patients were unable to identify triggers for their GA (93.2%, *n* = 68). The most common reported triggers were viral infections (2.74%, *n* = 2) and trauma (4.11%, *n* = 3). Half of the patients had comorbid conditions, most frequently atopic dermatitis (17.8%, *n* = 13), obesity (9.59%, *n* = 7), allergic rhinitis (6.85%, *n* = 5), and asthma (6.85%, *n* = 5). Although less common, some patients had diagnosed comorbid prediabetes (4.11%, *n* = 3), hypothyroidism (2.74%, *n* = 2), and diabetes mellitus (2.74%, *n* = 2). Of note, all cases of hypothyroidism and prediabetes were seen in patients with localized GA. Diabetes cases were split between subcutaneous and generalized GA. There were also single cases of comorbid systemic lupus erythematosus and hyperlipidemia in the localized GA subtype. Otherwise, there were no clear differences between GA subtypes with regard to their associated comorbidities or triggers.

The median duration of the disease was 11.00 months (interquartile range (IQR) 15.75 months). Generalized GA had the shortest duration (median 10.00 months and IQR 15.50 months), while subcutaneous GA had the longest duration (median 12.00 months and IQR 29.00 months). Topical steroids were the most common treatments for localized and generalized GA, used in 86.5% and 100.00% of cases, respectively. Topical immunomodulators (e.g., tacrolimus) were used in some cases of localized GA (9.62%, *n* = 5) and rarely in generalized and subcutaneous subtypes (11.1%, *n* = 1 and 8.33%, *n* = 1, respectively). Intralesional steroids were given to one of the nine patients in our cohort with generalized GA (11.1%), as well as to one patient with localized (1.92%) and one patient with subcutaneous (8.33%) GA subtypes.

Excision was utilized for 50% of cases of subcutaneous GA. The decision to pursue excision was made either due to an unclear diagnosis and/or the lesion causing the patient discomfort. Subcutaneous and generalized GA had the highest recurrence rates (45.5%, range: 3 months to 5 years post-excision and 33.3%, range: 1 month to 17 months post-initial assessment, respectively). Approximately 9.59% (*n* = 7) of patients across subtypes had persistent disease despite treatment, and 17.8% (*n* = 13) of patients were lost to follow-up.

## 4. Discussion

The study we present sheds further light on the growing body of knowledge regarding GA. Although GA is not uncommon, its etiology, presentation, and treatment are not entirely understood, particularly with regard to its rarer subtypes. Localized GA, the most common subtype, classically presents as flesh-colored or red papules that can coalesce into annular plaques [5]. Generalized GA constitutes about 15% of cases and presents with lesions with similar morphology to the localized variant, but it is usually defined as >10 lesions, although others have defined it as the involvement of the extremities and trunk [2, 8]. Subcutaneous GA is characterized by an asymptomatic, expanding nodule most commonly seen on the lower extremities [9]. The patch and perforating variants are less common and were not observed in our cohort but are characterized by erythematous patches on the trunk and proximal extremities or 3-4 millimeter papules in annular configuration often with central umbilication, respectively [3, 10]. Histologically, conventional GA exhibits palisading histiocytes around degenerated collagen, with increased dermal mucin. Subcutaneous GA has histologic features indistinguishable from rheumatoid nodules, with histiocytes arranged around necrobiotic collagen in the deep dermis or subcutaneous fat (Figures 3(b) and 3(c)) [11].

The pathogenesis of GA remains unclear. One popular hypothesis is that of primary necrobiosis secondary to trauma [5, 12]. This may be mediated by the release of nonspecific esterase, acid phosphatase, and lysosome by histiocytes. An alternative hypothesis is that of a delayed-type hypersensitivity reaction. It is believed that a T_H_1 reaction stimulates macrophages to produce matrix metalloproteinases, leading to connective tissue degradation, which is consistent with histopathological findings of focal collagen degradation [13]. Previous studies have found cytokines produced by T-helper cells, including TGF-*β* and IL-2, in lesions of patients with GA which further supports this hypothesis; however, studies on immunocompromised patients with GA have shown that the pathogenesis of this disease may be more complex [5]. It is unclear what proportion of GA patients have a preceding trigger. Prior reports have noted diverse triggers, including vaccines (e.g., hepatitis B, tetanus, and Bacillus Calmette–Guérin), viral infections (e.g., varicella, herpes zoster, and COVID-19), medications (e.g., allopurinol, amlodipine, and anti-TNF agents), trauma, arthropod bites, and malignancy [14]. Although most of our cases were idiopathic, approximately 7% of patients endorsed triggers. These included viral infections (including Epstein–Barr virus) and trauma (including arthropod bites), consistent with data in existing literature [14]. The variety of triggers may reflect GA's uncertain etiology, which could involve a type IV delayed-type hypersensitivity reaction to an unknown antigen [15].

A highly debated topic is whether GA is associated with other conditions, such as metabolic, autoimmune, and neoplastic disorders. One of the largest recent case-control studies (*n* = 5137 patients with GA, 51169 controls) used ICD-10 codes to demonstrate an association between GA and hyperlipidemia (adjusted odds ratio (aOR) 1.15, 95% confidence interval (CI) 1.08–1.23), hypothyroidism (aOR 1.59, 95% CI 1.14–1.22), systemic lupus erythematosus (SLE) (aOR 3.06, 95% CI 1.86–5.01), rheumatoid arthritis (aOR 2.05, 95% CI 1.34–3.13), and diabetes (aOR 1.67, 95% CI 1.55–1.80); however, this study was largely based on the adult population [4]. Although few large-scale pediatric GA studies exist, the cooccurrence of pediatric GA with type 1 diabetes mellitus, hematologic malignancy, and atopy has been documented in existing literature [3]. A recent case series further highlighted diabetes mellitus as well as thyroid disease and hyperlipidemia as comorbidities of pediatric GA, which was observed in our cohort in small numbers [16]. The prevalence of diabetes, specifically, in our cohort (2.74%, *n* = 2) was higher than that reported in youth in the general population (2.22 per 1,000 individuals) [17]. However, the association between pediatric GA and diabetes mellitus remains heavily debated [4, 6, 7, 18]. We had several patients with manifestations of atopy, notably atopic dermatitis (*n* = 13), allergic rhinitis (*n* = 5), and asthma (*n* = 5), which is in line with a recent pediatric GA retrospective study in which 23 of 47 patients included had evidence of atopy [18]. However, none of these atopic comorbidities were clearly associated with a particular GA subtype in our cohort. Furthermore, the relatively low sample size of our study and lack of a control group limit our ability to infer a clear association between GA and the comorbidities discussed.

Finally, the management of GA remains challenging. As seen in our study, steroids are often the most frequently prescribed treatment [19]. Yet, their efficacy is mixed, with some studies demonstrating rapid clearance and others finding improvement in only about half of GA patients [8, 19]. Treatment efficacy may be limited in part by the GA subtype. Whereas localized GA tends to be self-limited, other subtypes, particularly generalized GA, can have high recurrence rates. Many treatments have been investigated for generalized GA with inconsistent results and varied side effects, including topical and intralesional steroids, topical immunomodulators (tacrolimus), antimicrobials (doxycycline, dapsone, and hydroxychloroquine), phototherapy, TNF-alpha inhibitors, and oral vitamin E [19–21]. Of the patients in our cohort who pursued these therapies, namely, intralesional steroids or topical immunomodulators, there was no clear association with a particular outcome.

Although there is a growing body of evidence regarding GA, much remains to be elucidated. Further large-scale, prospective studies should be conducted to further characterize the various GA subtypes, their management, and associated comorbid conditions.

## Figures and Tables

**Table 1:** Patient and disease characteristics in pediatric GA (*N* = 73).

	All (*N* = 73)	Localized (*n* = 52)	Subcutaneous (*n* = 12)	Generalized (*n* = 9)
Sex, *n* (%)				
Female	46 (63.0)	30 (57.7)	10 (83.3)	6 (66.7)
Male	27 (37.0)	22 (42.3)	2 (16.7)	3 (33.3)
Ethnicity, *n* (%)				
Caucasian	39 (53.4)	27 (51.9)	7 (58.3)	5 (55.6)
African American	20 (27.4)	15 (28.8)	2 (16.7)	3 (33.3)
Hispanic	11 (15.1)	7 (13.5)	3 (25.0)	1 (11.1)
Unknown	3 (4.11)	3 (5.8)	0	0
Age at evaluation, years, mean (SD)^†^				
Affected sites, *n* (%)	6.98 (4.35)	6.67 (4.26)	7.17 (3.49)	8.56 (5.88)
Hands or feet	36 (49.3)	25 (48.1)	8 (66.7)	3 (33.3)
Legs	34 (46.6)	24 (46.2)	5 (41.7)	5 (55.6)
Arms	17 (23.3)	7 (13.5)	3 (25.0)	7 (77.8)
Trunk	8 (11.0)	2 (3.85)	0	6 (66.7)
Face	4 (5.48)	2 (3.85)	1 (8.33)	1 (11.1)
Mean affected sites/patient	1.36	1.15	1.42	2.44
Histopathology				
Yes	21 (28.8)	7 (13.5)	10 (83.3)	4 (44.4)
No	52 (71.2)	45 (86.5)	2 (16.7)	5 (55.6)
Symptoms, *n* (%)				
Pruritus	20 (27.4)	12 (23.1)	4 (33.3)	4 (44.4)
Pain	3 (4.11)	2 (3.85)	1 (8.33)	0
None	50 (68.5)	38 (73.1)	7 (58.3)	5 (55.6)
Triggers, *n* (%)				
Unknown	68 (93.2)	51 (98.1)	9 (75.0)	8 (88.9)
Viral infection	2 (2.74)	1 (1.92)	0	1 (11.1)
Trauma	3 (4.11)	0	3 (25.0)	0
Associated conditions, *n* (%)				
None	38 (52.1)	26 (50.0)	7 (58.3)	5 (55.6)
Atopic dermatitis	13 (17.8)	11 (21.2)	0	2 (22.2)
Obesity	7 (9.59)	5 (9.62)	1 (8.33)	1 (11.1)
Allergic rhinitis	5 (6.85)	3 (5.77)	1 (8.33)	1 (11.1)
Asthma	5 (6.85)	3 (5.77)	2 (16.7)	0
Prediabetes	3 (4.11)	3 (5.77)	0	0
Hypothyroidism	2 (2.74)	2 (3.85)	0	0
Diabetes	2 (2.74)	0	1 (8.33)	1 (11.1)
SLE^‡^	1 (1.37)	1 (1.92)	0	0
Hyperlipidemia	1 (1.37)	1 (1.92)	0	0
Treatments used				
None	7 (9.59)	4 (7.69)	3 (25.0)	0
Topical steroid	56 (76.7)	45 (86.5)	2 (16.7)	9 (100)
Topical tacrolimus	7 (9.59)	5 (9.62)	1 (8.33)	1 (11.1)
Excision	6 (8.22)	0	6 (50.0)	0
Intralesional steroid	3 (4.11)	1 (1.92)	1 (8.33)	1 (11.1)
Duration of disease, months, mean (SD)				
Outcome, *n* (%)	16.9 (17.1)	18.7 (19.4)	17.3 (17.2)	12.0 (9.68)
Clearance	44 (60.3)	29 (55.8)	7 (58.3)	8 (88.9)
Improvement	9 (12.3)	7 (13.5)	1 (8.33)	1 (11.1)
Persistence	7 (9.59)	5 (9.62)	2 (16.7)	0
Uncertain^§^	13 (17.8)	11 (21.2)	2 (16.7)	0
Recurrence rate (%)	24.2	17.4	45.5	33.3

^†^SD: standard deviation; ^‡^systemic lupus erythematosus; ^§^due to loss to follow-up.

## Data Availability

The data supporting the current study are available from the first author upon request.
